# Spin relaxation dynamics of holes in intrinsic GaAs quantum wells studied by transient circular dichromatic absorption spectroscopy at room temperature

**DOI:** 10.1038/s41598-017-00396-1

**Published:** 2017-03-21

**Authors:** Shaoyin Fang, Ruidan Zhu, Tianshu Lai

**Affiliations:** State Key Laboratory of Optoelectronic Materials and Technologies, School of Physics, Sun Yat-Sen University, Guangzhou, 510275 P.R. China

## Abstract

Spin relaxation dynamics of holes in intrinsic GaAs quantum wells is studied using time-resolved circular dichromatic absorption spectroscopy at room temperature. It is found that ultrafast dynamics is dominated by the cooperative contributions of band filling and many-body effects. The relative contribution of the two effects is opposite in strength for electrons and holes. As a result, transient circular dichromatic differential transmission (TCD-DT) with co- and cross-circularly polarized pump and probe presents different strength at several picosecond delay time. Ultrafast spin relaxation dynamics of excited holes is sensitively reflected in TCD-DT with cross-circularly polarized pump and probe. A model, including coherent artifact, thermalization of nonthermal carriers and the cooperative contribution of band filling and many-body effects, is developed, and used to fit TCD-DT with cross-circularly polarized pump and probe. Spin relaxation time of holes is achieved as a function of excited hole density for the first time at room temperature, and increases with hole density, which disagrees with a theoretical prediction based on EY spin relaxation mechanism, implying that EY mechanism may be not dominant hole spin relaxation mechanism at room temperature, but DP mechanism is dominant possibly.

## Introduction

Spin relaxation dynamics and related relaxation mechanism of carriers in semiconductors has been attracting much attention for the demands to understand fundamental spin physics and to develop spintronics. Spin relaxation dynamics of electrons has been studied extensively in bulk GaAs semiconductors^1–7^ and two-dimensional GaAs quantum wells^7–15^. It was found that spin relaxation time of electrons was dominated by the Dyakonov-Perel (DP) spin relaxation mechanism for both (001)-oriented bulk GaAs^1, 3, 7^ and two-dimensional quantum wells^10^, but the DP spin relaxation mechanism was suppressed so substantially in (110)-oriented GaAs quantum wells that spin relaxation time was prolonged significantly^14, 15^. In contrast to the extensive studies of spin relaxation of electrons, spin relaxation of holes in bulk GaAs and quantum wells was studied much less because spin relaxation of holes was much faster than one of electrons and usually hidden by spin relaxation of electrons. Less and controversial spin relaxation times of holes were reported, and a widely variable spin relaxation time ranging from subpicosecond to tens of picoseconds has been observed^16–22^. Hilton *et al.* observed that spin relaxation time of heavy holes in intrinsic bulk GaAs was about 110 fs at room temperature by means of non-degenerate pump-probe circular dichromatic absorption spectroscopy^16^. Damen *et al.* studied the spin relaxation dynamics of holes in n-doped GaAs quantum wells by analyzing the decay dynamics of circular polarization of photoluminescence in the range of low temperature^17^, and observed a spin relaxation time of 4 ps. Amand *et al.* investigated the spin relaxation dynamics of holes in intrinsic GaAs quantum wells at the low temperature of 1.7 K using time evolution of the ratio of circularly polarized light-excited to linearly polarized light-excited photoluminescence intensity^18^, and achieved a hole spin lifetime of 2.6 ps. However, a longer spin relaxation time of holes of tens of picoseconds was also reported at low temperature^19–22^. Spin relaxation times of holes in *p*-dope GaAs quantum wells were measured by means of circular photogalvanic effect of THz radiation^19^ and time-resolved Kerr rotation beat spectroscopy^20^. Longer spin relaxation times of heavy holes, respectively up to ~25 ps at 4 K^19^ and 60 ps at 6 K^20^, were reported. Bar-Ad *et al.* reported a longer hole spin lifetime of 35~50 ps in GaAs quantum wells at 4.2 K using degenerate pump-probe circular dichromatic absorption spectroscopy^21^. Ostatnický *et al.* studied the spin relaxation of holes within excitons in intrinsic GaAs quantum wells by non-degenerate pump-probe transmission spectroscopy, and also observed a longer hole spin lifetime of 30 ps at 4 K^22^, and confirmed the results reported by Bar-Ad *et al.* Consequently, one can find that there is a larger difference between spin relaxation times of holes reported even though they were measured all at low temperature, but the cause on the larger difference is incompletely clear so far. On the other hand, spin relaxation time of holes at room temperature has not been reported for GaAs quantum wells except for bulk GaAs^16^. However, it is very important for the development of spintronic devices. It is very demanded to study it. Furthermore, spin relaxation mechanism of holes was still unknown for GaAs quantum wells. It was studied theoretically only for bulk GaAs^23^. No experimental results are available on spin relaxation mechanism of holes for either bulk GaAs or quantum wells.

In this article, we study spin relaxation dynamics of holes in intrinsic GaAs quantum wells for the first time at room temperature using a simple degenerate circular dichromatic pump-probe absorption spectroscopy. A key empirical model is developed to enable extracting spin relaxation time of holes from transient circular dichromatic absorption dynamic data. Spin relaxation time of few picoseconds is obtained. Photoexcited hole density dependence of spin relaxation dynamics is studied, which reveals possible DP dominant spin relaxation mechanism of holes in intrinsic GaAs quantum wells at room temperature.

## Results and Discussion

### Ultrafast Dynamics of Hole Spin Relaxation and Thermalization of Nonthermal Carriers in Intrinsic GaAs Quantum Wells

**Figure 1:**
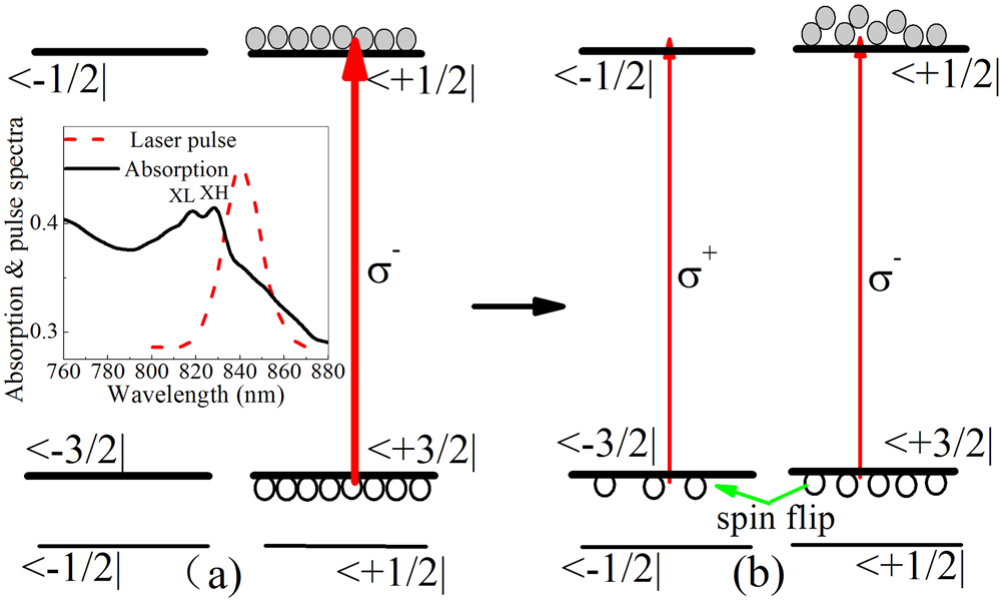
The schematic of band structure of intrinsic GaAs quantum wells. (**a**) Selective excitation of σ^−^ circularly polarized pump pulses. (**b**) Selective detection of σ^−^ and σ^+^ circularly polarized probes. The inset in (**a**) shows absorption spectrum of the sample and power spectrum of femtosecond laser pulses.

The schematic of band structure of intrinsic GaAs quantum wells is shown in Fig. 1. Absorption spectrum of the sample is measured experimentally and plotted in the inset of Fig. 1(a). The positions of XH (829 nm) and XL peaks correspond to the lowest energy (n = 1) of heavy- and light-hole excitons, respectively. Spin relaxation and transport dynamics of electrons for this sample has been studied previously^9, 10, 24^. Spin relaxation time ranging from 80 to 120 ps was observed at room temperature^9, 10^.

**Figure 2:**
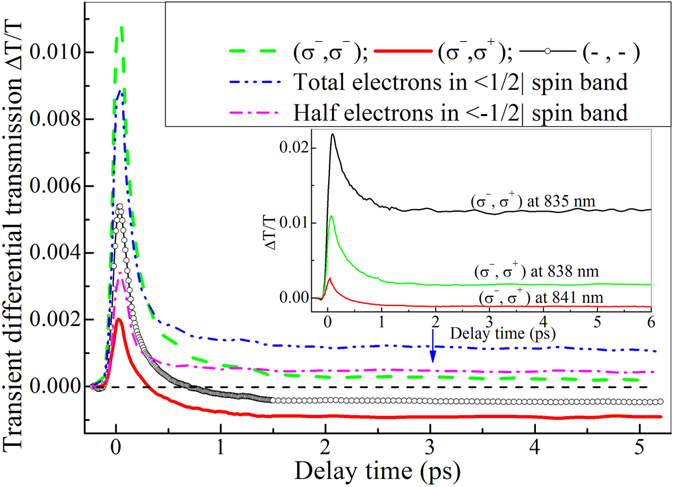
Transient differential transmissions for co-circularly (σ^−^, σ^−^) and cross-circularly (σ^−^, σ^+^) polarized as well as colinearly (−, −) polarized pump and probe pulses. They are measured at central wavelength of 841 nm with an excitation density of 8.4*10^9^ cm^−2^. In inset transient differential transmission for cross-circularly polarized (σ^−^, σ^+^) pump and probe is plotted for femtosecond laser at three different central wavelengths of 841, 838 and 835 nm.

To avoid the photo-excitation of light holes, the central wavelength of femtosecond pulses is tuned to 841 nm whose energy is lower than the lowest energy (XH) of heavy hole excitons, which is different from nearly resonant excitation of XH by 830 nm laser in ref. 10. The spectrum of femtosecond pulses is plotted together with the absorption spectrum in the inset of Fig. 1(a). One can see that photo-excitation of heavy holes should be overwhelming. Figure 1(a) shows the selective transition of spin-dependent electrons from |3/2> heavy-hole valence band excited by a left-handed circularly polarized pump pulse (σ^−^). Then, hole spin relaxation first occurs due to much faster spin relaxation rate than one of electrons, as the spin flip from |3/2> to |−3/2> spin valence band shows in Fig. 1(b). As a left- (σ^−^) or right-handed (σ^+^) circularly polarized probe pulse is used to monitor the transient differential transmission of the sample, the accumulation in |−3/2> spin band or decay in |3/2> spin band of holes should be able to be revealed. Figure 2 shows such transient differential transmissions, as the dashed (σ^−^, σ^−^) and thick solid (σ^−^, σ^+^) lines show. A transient differential transmission for colinearly polarized pump and probe (−, −) is also measured for comparison and plotted in Fig. 2 by open circle line. It is worth noting that here transient differential transmission mainly reflects the change of transient absorption coefficient, while the influence of transient reflectivity change can be ignored because our sample is thicker and transient differential transmission is much stronger than the change of transient reflectivity.

Obviously, those three transient traces in Fig. 2 have different dynamic behaviors although they all have a strong positive peak near zero delay point. They decay fast in a picosecond from the positive peak near zero time and then approach a plateau region occurring in several picoseconds (such as the vertical arrow at 3 ps), but the plateau has a different height which just reflects different contributions of spin-polarized electrons and holes. The occurrence of the plateau just reflects the end of spin relaxation of spin-polarized heavy holes in |3/2> spin valence band injected by σ^−^ pump pulses, but spin relaxation of electrons in |1/2> spin conduction band has not taken place because spin relaxation time of electrons in the sample is larger than 80 ps.^9, 10^ (σ^−^, σ^+^) trace has a lowest negative plateau, which just reflects the contribution of spin relaxation of heavy holes because σ^+^ probe can only detect the contribution of heavy holes in |−3/2> spin valence band due to optical selection rule. (−, −) trace has also a negative plateau, but higher than one of (σ^−^, σ^+^) trace, which just reflect the different contribution of spin polarized electrons and holes to the differential transmission because the difference between the traces (σ^−^, σ^+^) and (−, −) only comes from the contribution of electrons in |−1/2> spin conduction band. As a result, the contribution of excited electrons (half of total excited electrons) in |−1/2> spin conduction band can be obtained by subtracting (σ^−^, σ^+^) trace from (−, −) trace, as the dashed dot line shows in Fig. 2. Alone spin polarized electrons indeed contributes a positive plateau, different from the negative plateau induced by spin polarized holes. It is the sign difference between the negative plateau induced by the excited holes in |−3/2> valence band and the positive plateau by the excited electrons in |−1/2> conduction band that reflects the competition between band filling and many-body (such as bandgap renormalization (BGR) and Sommerfeld enhancement) effects. For holes, band filling effect is weaker than many-body effect. However, for electrons the result is just reverse. The relatively weak band filling of heavy holes may originate from a big density of state in heavy hole valence band because the density of state depends on *m* ^3/2^ (*m* denotes effective mass). For GaAs, effective mass of heavy holes is almost seven times one of electrons so that the density of state in heavy-hole valence band is almost eighteen times one in conduction band. (σ^−^, σ^−^) trace shows a positive plateau at several picoseconds, which just originates from the stronger band filling contribution of excited total electrons in |1/2> spin conduction band. Similarly, the transient contribution of alone the excited total electrons can be extracted by subtracting (σ^−^, σ^+^) trace from (σ^−^, σ^−^) trace, as the dashed dot dot line shows in Fig. 2. Comparing the dashed dot dot with the dashed dot lines, one can see that the excited total electrons in |1/2> conduction band indeed results in a higher positive plateau than the half of the excited total electrons in |−1/2> conduction band.

To further reveal pure heavy-hole origin of unique (σ^−^, σ^+^) dynamics measured at 841 nm, we have performed photon energy dependent dynamic measurement for (σ^−^, σ^+^) circularly polarized pump and probe under a constant total excitation density, as three solid lines show in the inset of Fig. 2. The negative plateau disappears and becomes positive plateau as photon energy increases or central wavelength decreases to 838 nm from 841 nm. The positive plateau rises further when central wavelength reduces down to 835 nm. The rise of the plateau with photon energy originates from the increase of excited electron and light-hole density, respectively in <−1/2| spin conduction band and in <1/2| spin light-hole valence band due to the excitation enhancement of the light-hole transition <1/2| → <−1/2| and excitation weakening of heavy-hole transition <3/2| → <1/2|. Therefore, those dynamic evolution with photon energy again confirms (σ^−^, σ^+^) dynamics measured at 841 nm originates from pure heavy-hole spin relaxation.

Next, we discuss possible origin of the positive peak near zero delay time in transient traces. For (−, −) trace, it is well known that the positive peak originates from state filling of pump-excited non-thermal carriers^25, 26^ and coherent artifact^27^. Its fast decay reflects the thermalization of the nonthermal carriers, that is the formation of Fermi distribution of carriers in conduction and valence bands. Similarly, for (σ^−^, σ^−^) trace, the positive peak near zero time delay has the same origin as one in (−, −) trace has, but its fast decay has one more additional channel of spin relaxation of holes from <3/2| to <−3/2| spin valence bands besides thermalization. For (σ^−^, σ^+^) trace, the positive peak near zero time may mainly result from the coherent artifact and a weak state filling of a little amount of residual excited nonthermal electrons in <−1/2| spin conduction band. Its fast decay mainly originates from spin relaxation of holes into <−3/2| spin valence band from <3/2| spin valence band and a weak thermalization contribution of the residual excited nonthermal electrons. The little amount of residual excited nonthermal electrons comes from impure circular polarization of σ^−^ pump and high energy tail in the spectrum of σ^−^ pump pulses. In other words, impure σ^−^ pump contains a small fraction of σ^+^ polarization which can excite a little amount of nonthermal electrons in <−1/2| spin conduction band and holes in <−3/2| spin valence band, while the high energy tail in the spectrum of σ^−^ pump pulses can excite a little amount of nonthermal electrons in <−1/2| spin conduction band and holes in <1/2| spin valence band (excitation of light-hole excitons XL). The two residual excitations result in the little amount of nonthermal electrons into <−1/2| spin conduction band. Therefore, the information on spin relaxation of holes is contained in the fast decay process of the positive peaks in both (σ^−^, σ^−^) and (σ^−^, σ^+^) transient traces. In principle, spin relaxation lifetime of holes can be extracted by quantitatively analyzing the fast decay process in (σ^−^, σ^−^) or (σ^−^, σ^+^) transient trace. However, actually the spin relaxation of holes can be more sensitively reflected in (σ^−^, σ^+^) than in (σ^−^, σ^−^) traces because the thermalized contribution of excited nonthermal electrons is much weaker in (σ^−^, σ^+^) than in (σ^−^, σ^−^) traces. As a result, (σ^−^, σ^+^) trace will be analyzed quantitatively below to extract the spin relaxation time of holes.

### Modeling Ultrafast Dynamics of Hole Spin Relaxation and Thermalization of Nonthermal Carriers in Intrinsic GaAs Quantum Wells

An appropriate model needs developing quantitatively to extract the spin relaxation time of holes. Based on the above discussions, (σ^−^, σ^+^) trace can be described by the following model,

1 $$S(t)=aC(t)+C(t)\otimes (b{e}^{-t/{\tau }_{th}}+H(t/{\tau }_{s}))$$

where *C*(*t*) denotes the area-normalized cross-correlation function of pump and probe pulses and can be measured experimentally. Symbol ⊗ denotes the operation of convolution.

**Figure 3:**
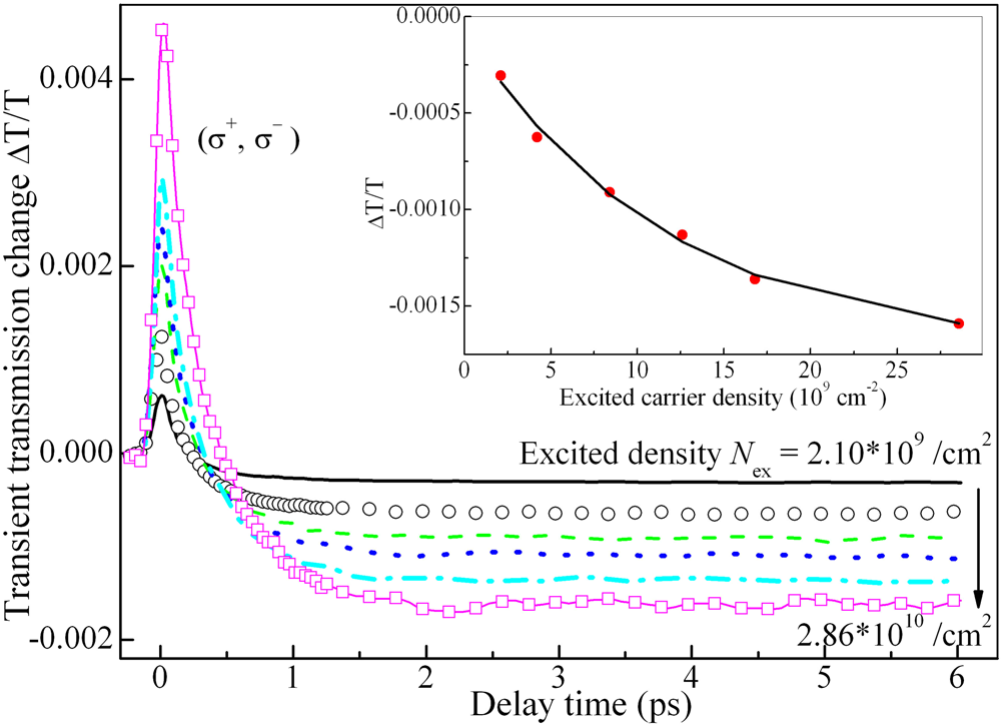
Ultrafast dynamics with cross-circularly polarized pump and probe as a function of excited hole density. In inset, the amplitude (filled circles) of transient differential transmission at ~3 ps versus excited hole density is plotted. Solid line is the best fitting to scattered filled circles with Eq. (2) described in text.

The first term in Eq. (1) describes coherent artifact approximately. The second denotes thermalized contribution of the residual excited nonthermal electrons, while *τ*_th_ describes the thermalized time of nonthermal electrons and can be extracted accurately by best fitting (−, −) transient trace. *H*(*t*/*τ*_s_) describes the dynamics of spin relaxation of holes, while *τ*_s_ is just the spin relaxation time of holes. The key problem is the specific form of *H*(*t*/*τ*_s_). As discussed above, pure holes can induce band filling and many-body effects simultaneously. The contribution of many-body effect to transient differential transmission is negative, while one of band filling is positive. The former is stronger than the latter in absolute amplitude so that total differential transmission is negative, as the negative plateau at a few picoseconds in (σ^−^, σ^+^) trace shows in Fig. 2. Therefore, *H*(*t*/*τ*_s_) should be a sum of two components formally. One component describes the contribution of band filling, while the other component does one of many-body effect. However, the difficulty is that we don’t know how much the weight of each component is, and thus it is difficult to write out the specific form of *H*(*t*/*τ*_s_). Nevertheless, we can know the total sum of the two components experimentally, as the negative plateau at a few picoseconds in (σ^−^, σ^+^) trace shows in Fig. 2. Furthermore, hole density dependence of the total sum can be obtained experimentally if pump intensity dependent experiments are carried out. Such experiments have been performed. (σ^−^, σ^+^) transient traces have been plotted in Fig. 3 for different excited hole density. The ampltiude of the plateau at 3 ps is taken as the total sum of hole-induced band filling and many-body effects, and is plotted in the inset of Fig. 3 by filled circles as a function of excited hole density. Physically, the total sum should approach to zero if excited hole density decreases down to zero. Contrarily, it should approach some saturation value if excited hole density is increasing progressively. Therefore, a justified formula, *y* = *c*(exp(−*p*/*P*_c_) − 1), is used to fit the total sum of excited hole-induced effects, as the solid line shows in the inset of Fig. 3. One can see the solid line fits those filled circles very well. The best fitting gives out the specific expression of the total sum of hole-induced effects as,

2 $$H(t/{\tau }_{s})=0.00173({e}^{-p(t/{\tau }_{s})/{P}_{c}}-1)$$

where *P*_c_ is a critical hole density for hole-induced transient absorption saturation, and is given as *P*_c_ = 1.17 × 10^10^/cm^2^ by the fitting.

Similar to electron spin relaxation^8^, based on rate equation of hole spin relaxation and negligible recombination of holes due to very long recombination time, the time evolution equation of hole density in |−3/2> spin valence band can be expressed by,

3 $$p(t/{\tau }_{s})=\frac{{P}_{0}}{2}(1-{e}^{-2t/{\tau }_{s}})$$

where *P*_0_ denotes total hole density excited by a σ^−^ pump pulse.

Substituting Eqs (2) and 3 into Eq. (1), a complete dynamic model to describe the dynamics of (σ^−^, σ^+^) transient trace is obtained as,

4 $$S(t)=aC(t)+C(t)\otimes (b{e}^{-t/{\tau }_{th}}+0.00173(\exp (-{P}_{0}(1-{e}^{-2t/{\tau }_{s}})/(2{P}_{c}))-1))$$

**Figure 4:**
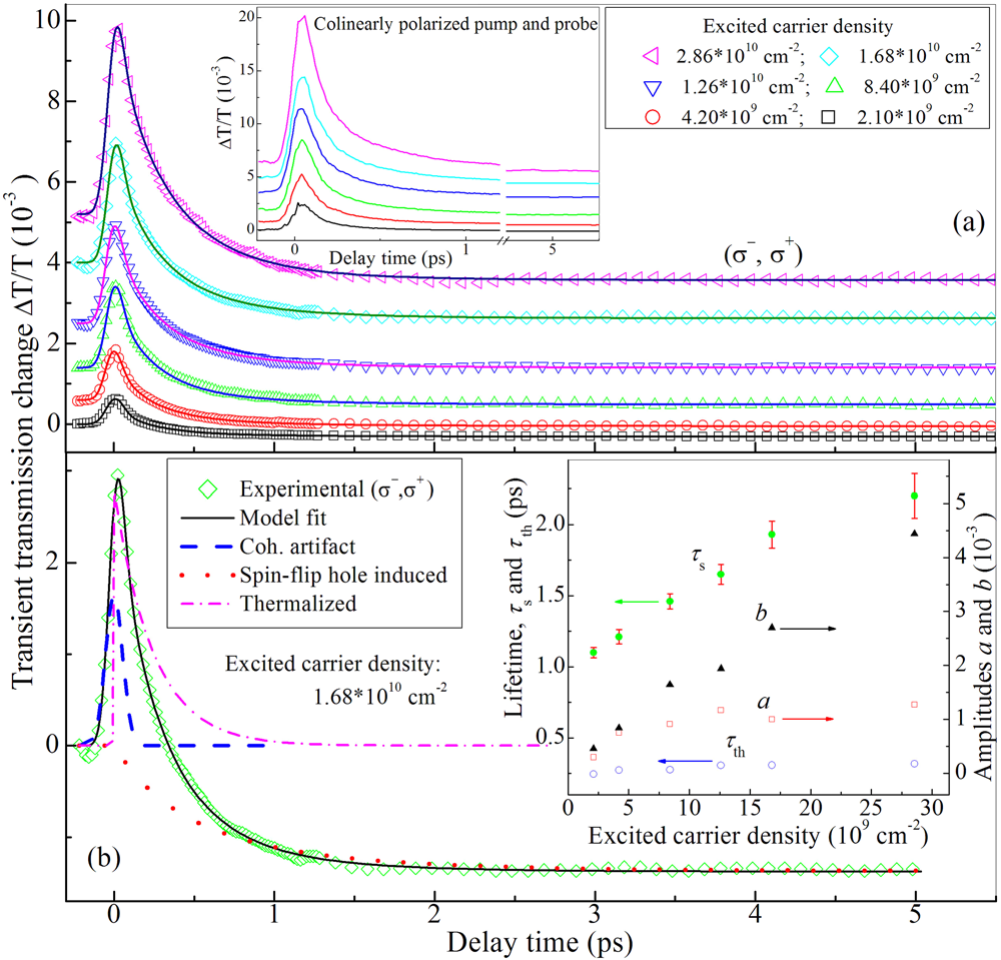
(**a**) (σ^−^, σ^+^) transient differential transmission (scattered symbols) for different excited hole density indicated in legend at room temperature. Solid lines are the best fitting to the scattered symbols with Eq. (4) in text. Inset: colinearly polarized transient differential transmission with the same excited hole density as one in (**a**) for the extraction of thermalization time *τ*_th_. (**b**) (σ^−^,σ^+^) transient differential transmission (scattered symbols, experimental data; solid line, best fitting) with the excited carrier density of 1.68 × 10^10^ cm^−2^ and its separation of three components: coherent artifact (dashed line), thermalization (dashed dot line) and spin-relaxed hole induced contribution (dot line). Inset: hole spin relaxation time *τ*_s_ (filled circles) plus error bars, carrier-thermalized time *τ*_th_ (open circles), the amplitudes of coherent artifact (open squares, *a*) and thermalization (triangles, *b*) components versus excited hole density. Errors of *τ*_th_, *a* and *b* are too less to be shown, and 5% of their values at most.

Equation (4) is used to fit all (σ^−^, σ^+^) transient traces with different hole densities (*P*_0_) excited by σ^−^ pump pulses. The experimental data (scatterred points) and best fittings (solid lines) are plotted in Fig. 4(a). One can see all fittings agree very well with experimental data.

One fitting example, showing separated three components: coherent artifact, thermalizing and spin-relaxed hole induced contributions, is plotted in Fig. 4(b) for the excited density of 1.68 × 10^10^ cm^−2^. The dashed line shows the coherent artifact which is an even symmetric peak near zero time. The dashed dot line indicates thermalizing dynamics of residual excited electrons and light holes. The dot line shows the spin relaxation dynamics of excited heavy holes. It is worth stressing that the decay time (*τ*_th_) of the thermalizing dynamics is extracted priorly by fitting (−, −) transient trace shown in the inset of Fig. 4(a) with corresponding excited density to (σ^−^, σ^+^) transient trace. Consequently, there are only three free parameters, *a*, *b* and *τ*_s_ in fitting to (σ^−^, σ^+^) transient traces due to prior determination of *τ*_th_. This can ensure the unique solution of parameters, *a*, *b* and *τ*_s_. The extracted four parameters, *a*, *b*, *τ*_s_ and *τ*_th_, are plotted as a function of excited carrier density in the inset of Fig. 4(b). One can see *τ*_s_ (filled circles) is obviously longer than *τ*_th_ (open circles). They are separated well. Parameters, *a* (open squares) and *b* (filled triangles), are also plotted as a function of excited carrier density in the inset of Fig. 4(b). One can see *b* is always stronger than *a*. *b* enhances almost linearly with excited carrier density, but *a* grows slowly and nonlinearly. Such an increase trend should be reasonable because coherent artifact is nonlinear effect, while the strength of thermalization is linearly dependent on pump-injected carrier density.

It is worth emphasizing that our *τ*_s_ is the first experimental report of hole spin relaxation time in GaAs/AlGaAs quantum wells at room temperature. Our *τ*_s_ agrees very well with reported spin lifetime of 110 fs in bulk GaAs at room temperature^16^ with consideration of the splitting of heavy- and light-hole valence bands in GaAs quantum wells. Taking account for weakening of spin-phonon scattering at low temperature, our *τ*_s_ also agrees well with reported hole lifetimes of 25–60 ps at low temperature of several Kelvins^19–22^, but disagrees with spin relaxation time of a few picoseconds reported at low temperature^17, 18^.

### Spin Relaxation Mechanism of Holes

The mechanism leading to spin relaxation of holes has been an open issue for either bulk GaAs or GaAs quantum wells, and was studied rarely. Shen *et al.* ^23^ studied the effects of DP and Elliott-Yafet (EY) mechanisms on spin relaxation of holes in intrinsic and p-doped bulk GaAs theoretically, and found that spin relaxation time of holes strongly depended on hole density and lattice temperature. For intrinsic GaAs, EY mechanism was predicted to be dominated at low temperature, while DP mechanism could contribute markedly at middle temperature range and in low hole density. At room temperature, it was predicted that spin relaxation time of holes decreased with increasing hole density according to EY mechanism. However, our measured *τ*_s_ increases, instead of decreases with the increase of excited hole density, implying that additional mechanism besides EY, such as DP mechanism, may become dominant in GaAs quantum wells. Actually, it was already predicted and proven experimentally that a spin relaxation time of electrons increased with excited electron density in intrinsic GaAs quantum wells according to DP mechansim^10^. Therefore, we tend to the viewpoint that DP mechanism may be still dominant for spin relaxation of holes in intrinsic GaAs quantum wells at room temperature and in the range of low and middle hole density. Further theoretical and experimental studies on spin relaxation mechanism of holes are expected for bulk GaAs and GaAs quantum wells.

## Methods

### Sample

The sample of two-dimensional quantum wells studied consists of 11 periods of 6 nm thick intrinsic GaAs wells separated by 10 nm thick Al_0.3_Ga_0.7_As barriers, grown on semi-insulating GaAs substrate by molecular beam epitaxy along the (001) direction (z-axis). The substrate is removed by polishing first and then selective chemical etching for transmission measurements. The substrate-free GaAs/AlGaAs quantum wells film is mounted on a piece of sapphire window by Van der Waals bonding.

### Measurement of Transient Circular Dichromatic Differential Transmission

Time-resolved degenerate circular dichromatic absorption spectroscopy is used here to study spin relaxation dynamics of heavy holes at room temperature. The experimental setup is very similar to one shown in ref. 8. A home-made self-mode-locked Ti: sapphire oscillator generates a train of femtosecond pulses with a repetition rate of 94 MHz, a linear polarization ratio larger than 200, the pulse duration of ~80 fs and a tunable central wavelength from 750 to 850 nm. The linear polarization ratio of femtosecond pulses is still larger than 100 after the pulses go through standard Michelson interferometer-type pump-probe setup. Otherwise, polarizers with higher extinction ratio are used necessarily. Two achromatic wideband quarter-wave plates are inserted into pump and probe paths to generate left- and right-handed circularly polarized pump and probe beams. Pump and probe is focused to a same area on the surface of sample with a spot size of ~50 μm in diameter. The differential transmission of the probe is measured by a silicon photodiode whose output signal is measured by a lock-in amplifier. An optical chopper modulates pump beam at a frequency of ~1.13 kHz and synchronizes the lock-in amplifier.
